# MiR-181a-driven downregulation of cholesterol biosynthesis through SREBP2 inhibition suppresses uveal melanoma metastasis

**DOI:** 10.1186/s13046-025-03459-8

**Published:** 2025-07-19

**Authors:** Rui Wang, Claudia Gilbert, Houda Tahiri, Chun Yang, Solange Landreville, Pierre Hardy

**Affiliations:** 1 Department of Pharmacology and Physiology, Université de Montréal, Montréal, QC H3T 1C5 Canada; 2 Research Center of CHU Sainte-Justine, Université de Montréal, Montréal, QC H3T 1C5 Canada; 3 Department of Ophthalmology and Otorhinolaryngology-Cervico-Facial Surgery, Faculty of Medicine, Université Laval, Quebec City, QC G1V 0A6 Canada; 4 Hôpital du Saint-Sacrement, Regenerative Medicine Division, CHU de Québec-Université Laval Research Centre, Quebec City, QC G1S 4L8 Canada; 5 Université Laval Cancer Research Center, Quebec City, QC G1R 3S3 Canada; 6 Centre de Recherche en Organogénèse Expérimentale de l’Université Laval/LOEX, Quebec City, QC G1J 1Z4 Canada; 7 Department of Pediatrics, Université de Montréal, Montréal, QC H3T 1C5 Canada

**Keywords:** Uveal melanoma, miR-181a, Metastasis, Cholesterol biosynthesis, SREBP2, Crizotinib, Combinational therapy

## Abstract

**Background:**

uveal melanoma (UM) is the most common primary intraocular tumor in adults, with metastasis being the leading cause of death. However, effective treatments for metastatic UM remain limited. Emerging evidence suggests that cholesterol metabolism plays a role in cancer progression, but its impact on UM metastasis is not well understood.

**Methods:**

we investigated the effects of miR-181a on UM metastasis using multiple UM cell lines and a suprachoroidal injection mouse model. Functional assays, including migration, invasion, and cancer stem-like cell (CSC) formation, were performed. The target of miR-181a was identified through bioinformatics, luciferase assays, and western blotting. Cholesterol levels were measured, and in vitro and in vivo studies assessed the therapeutic potential of combining miR-181a with crizotinib.

**Results:**

miR-181a significantly decreases UM cell migration, invasion, and metastasis. Mechanistically, miR-181a was found to target sterol regulatory element-binding protein 2 (SREBP2), thereby inhibiting cholesterol biosynthesis. This decrease in cholesterol levels hindered reduced epithelial-to-mesenchymal transition (EMT) and led to a decline in cancer stem-like cell (CSC) populations in UM. Furthermore, elevated cholesterol or overexpression of SREBP2 abrogated the anti-metastatic effects of miR-181a. Additionally, a combination of miR-181a and crizotinib significantly inhibited metastasis, both in vitro and in vivo.

**Conclusions:**

miR-181a inhibits UM metastasis by targeting SREBP2 and reducing cholesterol biosynthesis. Its combination with crizotinib may provide a promising therapeutic strategy for metastatic UM.

**Supplementary Information:**

The online version contains supplementary material available at 10.1186/s13046-025-03459-8.

## Introduction

Uveal melanoma (UM), the most prevalent primary intraocular malignancy in adults, originates from melanocytes located in the uveal tract of the eye [1]. Although therapeutic strategies, including enucleation, radiotherapy, and phototherapy, can effectively control localized tumors [2], approximately 50% of patients with UM develop metastases to distant organs, even after complete surgical removal of the primary tumor [3, 4]. Metastasis is a primary and direct contributor to mortality in individuals with UM [5]. Previous research in our laboratory has demonstrated that miR-181a suppresses the growth of UM cells [6]. This finding prompted us to investigate whether miR-181a might play a role in inhibiting UM metastasis.

Epithelial-to-mesenchymal transition (EMT) and cancer stem cells (CSCs) are critical drivers of cancer cell metastasis [7]. A key mechanism of metastasis is the EMT, which enhances cellular plasticity and facilitates the acquisition of stem cell-like characteristics by cancer cells, thus allowing CSCs to migrate and seed into new distant sites [8]. Furthermore, both EMT and CSCs are intricately linked to cholesterol metabolism in cancer. Previous research has demonstrated that multicellular tumor spheroids of human UM upregulate genes associated with cholesterol biosynthesis [9]. Elevated levels of cholesterol biosynthesis-associated proteins have been observed in mammospheres, and increased cholesterol biosynthesis is a defining characteristic of breast CSCs [10]. Additionally, a substantial body of evidence highlights positive correlations among cholesterol biosynthesis, esterification, and cancer metastasis [11–13]. Rapidly proliferating cancer cells exhibit an increased demand for cholesterol, which is meet through either upregulated cholesterol biosynthesis pathways or enhanced cholesterol uptake [14]. Interestingly, miR-181a has been shown to decrease total cholesterol levels in mice [15]. Profiling of small non-coding RNAs has identified miR-181a as a critical mediator of the inhibition of cholesterol biosynthesis in triple-negative breast cancer [16]. Moreover, the miRNA database suggests that miR-181a might potentially target sterol regulatory element-binding protein 2 (SREBP-2, coded by the *SREBF2* gene), a key regulator of cholesterol biosynthesis [17]. SREBP2 not only promotes stem cell-like properties but also contributes to cancer metastasis in prostate cancer and hepatocellular carcinoma [18, 19].

In addition, metastatic UM commonly localizes to the liver, lungs, kidneys, brain, and bones, primarily because the expression of c-Met is elevated in UM cells, and the organs express the c-Met ligands hepatic growth factor (HGF) [20]. Signaling pathways activated by c-Met have been associated with enhanced cell migration in UM and are believed to play critical roles in the metastatic progression of this disease [21]. Studies have suggested that crizotinib, an inhibitor of c-Met, may serve as a potential adjuvant therapy for patients with high metastatic risk [22].

Herein, we observed that miR-181a inhibited the migration of UM cells and metastasis in UM xenografted animals by reprogramming cholesterol biosynthesis through SREBP2 inhibition−a process involving EMT and CSCs for the first time. Combination therapy with miR-181a and crizotinib significantly impeded UM metastasis. These findings may provide a novel approach for the treatment of UM metastasis.

## Materials and methods

### Cell lines and culture

OMM2.5 cells (a gift from Pr. Bruce R. Ksander, Harvard University) were cultured in RPMI 1640 medium (Gibco, USA) supplemented with 10% FBS (Wisent, Canada), 1% penicillin-streptomycin (Wisent, Canada), 1% non-essential amino acids (Wisent, Canada), and 0.05 mM 2-mercaptoethanol (Gibco, Canada) [23]. UM001 cells (a gift from Dr. Solange Landreville, Université Laval) were grown in RPMI 1640 (Wisent, Canada) with 10% FBS, 1% non-essential amino acids, 1% HEPES (Gibco, Canada), and 0.5% penicillin-streptomycin [24]. Mµ2F cells were cultured in RPMI 1640 (Wisent, Canada) with 10% FBS and 16 µg/mL gentamycin [25]. Mel285 cells were maintained in RPMI 1640 (Wisent, Canada) with 10% FBS, 2 mM L-glutamine (Wisent, Canada), and 1% penicillin-streptomycin [26] OMM2.5, UM001, and Mµ2F cells were derived from patient liver metastasis [23–25], whereas Mel285 was derived from primary UM [26] Retinal pigment epithelia (RPE)-19 cells (purchased from ATCC) and 293T cells (purchased from ATCC) were cultured in DMEM (Gibco, Canada) with 10% fetal bovine serum (FBS, Wisent, Canada) and 1% penicillin-streptomycin (Wisent, Canada) [27]. All cell lines were cultured at 37 °C in a humidified incubator with 5% CO2. They were confirmed to be mycoplasma-free and were authenticated by short tandem repeat DNA profiling analysis.

### Wound-healing scratching assays

The wound-healing scratch assays were conducted as previously described [28]. Briefly, UM cells (5 × 10^4^ cells per well) were seeded into 24-well plates and allowed to reach confluence over 48 h. A straight-line scratch was made with a sterile 1,000 µL micropipette tip. After removal of debris with a PBS wash, RPMI1640 medium was added to the wells. Progress in wound closure was monitored and photographed at specified time points with an inverted phase-contrast microscope (Incucyte^®^ SX5).

### Transwell invasion assays

Transwell invasion assays were performed as previously described [28]. Briefly, UM cells were pre-incubated for 48 h before seeding. After trypsinization, the cells were washed with PBS and counted with the trypan blue exclusion method. A total of 5 × 10^5^ viable cells were placed in the upper chamber (8 μm, Sarstedt, Canada) in 24-well plate (Sarstedt, Canada), which had been pre-coated with Matrigel (BD Biosciences, San Jose, CA). The lower chambers were filled with RPMI1640 medium supplemented with 10% FBS to act as a chemoattractant. After 24 h, the non-migrated cells on the upper surface were removed with a cotton swab. Cells that had migrated or invaded into the bottom of the inserts were fixed in 3% paraformaldehyde, stained with 0.5% crystal violet (Sigma, United States), and allowed to dry. The cells in three randomly selected microscopic fields were then photographed and counted with an inverted phase-contrast microscope (DMi1, Leica).

### Animal studies

Animal experiments were approved by the CHU Sainte-Justine Animal Care Committee (Protocol 2021–2820). A mouse model of metastasis was generated using suprachoroidal injection of UM cells. In brief, 2 × 10^6^ OMM2.5-luciferase (Luc) cells in 3 µL PBS were injected suprachoroidally into the 4- to 6-week-old NOD.Cg-*Prkdc*^*scid*^*Il2rg*^*tm1Wjl*^/SzJ (NSG) mice (The Jackson Laboratory, Montreal, Canada) under anesthesia. One week later, the mice were intravitreally administered with 2 µg miR-NC/miR-181a (Thermo Fisher Scientific) twice per week for 3 weeks or treated orally by gavage with vehicle (water) or crizotinib at 50 mg/kg (MedChemExpress, USA) daily 5 days per week for 8 weeks. In vivo bioluminescence imaging of metastasis was performed weekly in isoflurane anesthetized mice with an IVIS Lumina II instrument (Perkin Elmers, USA). Enucleation of injected eyes were performed at day 28. At the endpoint, organs including the liver, lungs, bones, kidneys, lymph nodes and brain were harvested for bioluminescence imaging. Freshly dissected eyes and livers were embedded in Tissue-Tek O.C.T. compound (Sakura), frozen in ice-cold isopentane, and stored at -80 °C for immunoblotting and immunohistochemistry.

### Immunofluorescence staining

Freshly dissected tissues were embedded in Tissue-Tek O.C.T. compound (Sakura), frozen in ice-cold isopentane, and stored at -80 °C. For cryosectioning, the frozen blocks were equilibrated to -20 °C in the cryostat chamber before being cut into 4 μm sections with CryoStar NX50 Cryostat (Thermo Fisher Scientific, United States). The sections were mounted on coated glass slides, dried, and stored at 4 °C until further use. Tumor sections were blocked with 1% bovine serum albumin and incubated with primary antibodies overnight at 4 °C, then incubated with secondary antibodies for 1 h at room temperature. The primary and secondary antibodies were used at the recommended dilutions, as listed in Table S2. Cell nuclei were stained with DAPI (Sigma, Canada). Fluorescent images were acquired by confocal laser scanning microscopy (LSM780, Carl Zeiss) at ×20 magnification with three Z-stacks at 1-µm intervals and constant exposure settings. GP100 fluorescence positive cells was quantified by ImageJ.

### RNA isolation and quantitative real-time PCR

Total RNA was extracted from cultured cells with TRIzol reagent (Thermo Fisher Scientific), treated with DNase I (Ambion), and reverse transcribed with the iScript Reverse Transcription Supermix (Qiagen). Quantitative PCR (qPCR) was performed with the miRCURY LNA SYBR Green Master Mix (Qiagen) with primers listed in Table S1. All qPCR reactions were conducted on a StepOnePlus™ Real-Time PCR System (Applied Biosystems), and data were analyzed in StepOne Software Version 2.1. Each qPCR experiment was performed at least three times using different RNA samples from separate knockdown experiments, and the results represent the average of all experiments. The mRNA fold change was calculated according to the threshold cycle (Ct) with the formula 2 − Δ(ΔCt), where ΔΔCt = [(Ct _target gene / miR− 181a_ - Ct _GAPDH / miR− 103_) in treated cells] - [(Ct _target gene / miR− 181a_ - Ct _GAPDH / miR− 103_) in control cells].

### Western blotting

Homogenized tissues or cells were lysed in RIPA Lysis and Extraction Buffer (Thermo Fisher Scientific) containing phosphatase and protease inhibitor cocktails for 30 min on ice, then sonicated for 15 s. The lysates were then centrifuged, and the supernatants were collected for immunoblotting. Protein concentrations were determined with a Pierce™ BCA Protein Assay Kit (Thermo Fisher Scientific). Thirty micrograms of protein per sample were loaded into each well and separated by sodium dodecyl sulfate polyacrylamide gel electrophoresis (SDS-PAGE). The protein blots were incubated with immunoglobulin G primary antibodies overnight at 4 °C. The following day, after three washes with a Tris Buffered Saline with Tween 20 (TBST) buffer, the blots were incubated with anti-IgG peroxidase-conjugated secondary antibodies for 1 h at room temperature. After three additional washes with TBST buffer, detection was performed with the Clarity Max ECL substrate (Bio-Rad) and the chemiluminescent imaging system (Bio-Rad, United States). Antibodies used for the immunoblotting assays are listed in Table S2.

### Spheroid formation assays

Melanosphere formation assays were conducted as previously described [28]. Forty-eight hours after treatment, UM cells were collected, washed with PBS, and plated in ultralow-attachment 96-well plates (Thermo Fisher Scientific) at a density of 5,000 cells per well in DMEM/F12 medium supplemented with 1 mL B27, 10 ng/mL bFGF, and 20 ng/mL EGF. After 7 days, the melanospheres were collected, dissociated into single cells, and replated at a density of 5,000 cells per well for secondary and tertiary melanosphere formation rounds. The melanospheres area size was measured by Incucyte S3 Live Cell Analysis Instrument (Sartorius, Germany) on day 7 after each replating.

### Aldehyde dehydrogenase assays

The aldehyde dehydrogenase (ALDH) activity was assessed with the ALDEFLUOR™ kit (Stem Cell Technologies, Vancouver, BC, Canada) according to the manufacturer’s instructions. In brief, UM cells were treated with miR-181a for 48 h, then suspended in ALDEFLUOR™ assay buffer containing ALDEFLUOR™ reagent (BODIPY-Aminoacetaldehyde), with or without the addition of the ALDEFLUOR™ DEAB reagent (diethylaminobenzaldehyde, a specific inhibitor of the ALDH1 enzyme, used as a control). After a 50-minute incubation at 37 °C, the cells were washed, resuspended in ALDEFLUOR™ assay buffer, and analyzed with a BD FACS Canto II flow cytometer (BD, United States).

### RNA-sequencing analyses

Total RNA was quantified, and its integrity was assessed with a LabChip GXII (PerkinElmer) instrument. Libraries were generated from 250 ng of total RNA as follows: mRNA enrichment and library preparation were performed with an Illumina Stranded mRNA Prep Kit (Illumina), according to the manufacturer’s recommendations. Libraries were quantified with a KAPA Library Quantification Kits−Complete kit (Universal) (Kapa Biosystems). Average fragment size was determined with a Fragment Analyzer (Agilent). The libraries were normalized and pooled, then denatured in 0.02 N NaOH and neutralized with HT1 buffer. The pool was loaded at 175pM on an Illumina NovaSeq S4 lane with the Xp protocol, according to the manufacturer’s recommendations. The run was performed for 2 × 100 cycles (paired-end mode). A phiX library was used as a control and mixed with libraries at a 1% level. Base calling was performed with RTA v3. The program bcl2fastq2 v2.20 was then used to demultiplex samples and generate fastq reads. Bioinformatics analyses were performed at the Bioinformatics core facility of the Montreal Clinical Research Institute (IRCM).

### Cholesterol measurement

Total cholesterol was determined with a commercially available assay kit (Abcam; ab65359). Briefly, after treatment, cells and tissues were harvested and washed with cold PBS. Cholesterol was extracted by resuspension of the samples in 200 µL chloroform: isopropanol: NP-40 mixture (7:11:0.1) with a micro-homogenizer. The extract was centrifuged at 15,000 × g for 10 min, and the organic phase (liquid) was carefully transferred to a new tube by avoiding the pellet. The solvent was air-dried at 50 °C to remove chloroform, then vacuum dried for 30 min to eliminate any residual organic solvent. The dried lipids were dissolved in 200 µL of Assay Buffer II/Assay Buffer, with sonication or vortexing to facilitate dissolution. Cholesterol levels were then measured according to the OD570 nm after incubation with the total cholesterol reaction mix.

### Analysis of miR-181a and SREBF2 expression and prognosis in datasets

The RStudio analysis platform was employed to assess the expression and prognostic significance of miR-181a and SREBF2 in TCGA datasets. To ensure data quality, only miRNAs with a mean count greater than 1 were retained, and 68 patients with complete clinical information were included in the survival analysis. SREBF2 transcript levels were evaluated across spindle, mixed, and epithelioid UM subtypes based on histological data from 80 primary UM patients in TCGA. The association between miR-181a and SREBF2 expression and UM prognosis was investigated using the PROGgeneV2 tool and gene expression data from 65 primary UM patients in the GEO dataset GSE22138.

### Dual luciferase assays and plasmid construct

The 3’UTR-binding sites of *SREBF2* mRNA and miR-181a were identified with online tools (miRSystem, targetscan, and miRmap). The wild-type and mutated *SREBF2* 3’UTR sequences (Integrated DNA Technologies) were inserted into the pmirGLO vector (Promega, Madison, WI, USA). Subsequently, 293T cells were co-transfected with the pmir-wt-*SREBF2* vector and miR-33a mimics or a mimic-negative control (NC), or with the pmir-mut-*SREBF2* vector and miR-33a mimics or NC. After 48 h of transfection, luciferase activity was measured with the Dual-Luciferase Reporter Assay System (Promega), according to the manufacturer’s guidelines. Data are presented as relative light units (RLU), determined as the luminescence of firefly luciferase divide by that of Renilla luciferase. Luminescent signals were detected using the CLARIOstar system (BMG LABTECH, Germany). For the overexpression of SREBP2 and AKT3 during transfection, we utilized 3′UTR-deficient pcDNA3.1-based plasmids (pcDNA3.1-SREBP2 and pcDNA3.1-AKT3) to ectopically express the respective genes in miR-181a-transfected UM cells.

### Statistical analysis

Statistical analysis was conducted with two-tailed Student’s t-tests for comparisons between two groups, and one-way or two-way analysis of variance (ANOVA) with multiple testing correction for comparisons among multiple groups. GraphPad Prism 10 (https://www.graphpad.com/features) was used to determine statistical significance. Except for RNA-seq, all experiments were repeated at least three times, and the results are presented as mean ± SEM. A *P*-value of less than 0.05 was considered statistically significant.

## Results

### MiR-181a restricts UM metastasis in vitro and in vivo

**Fig. 1:**
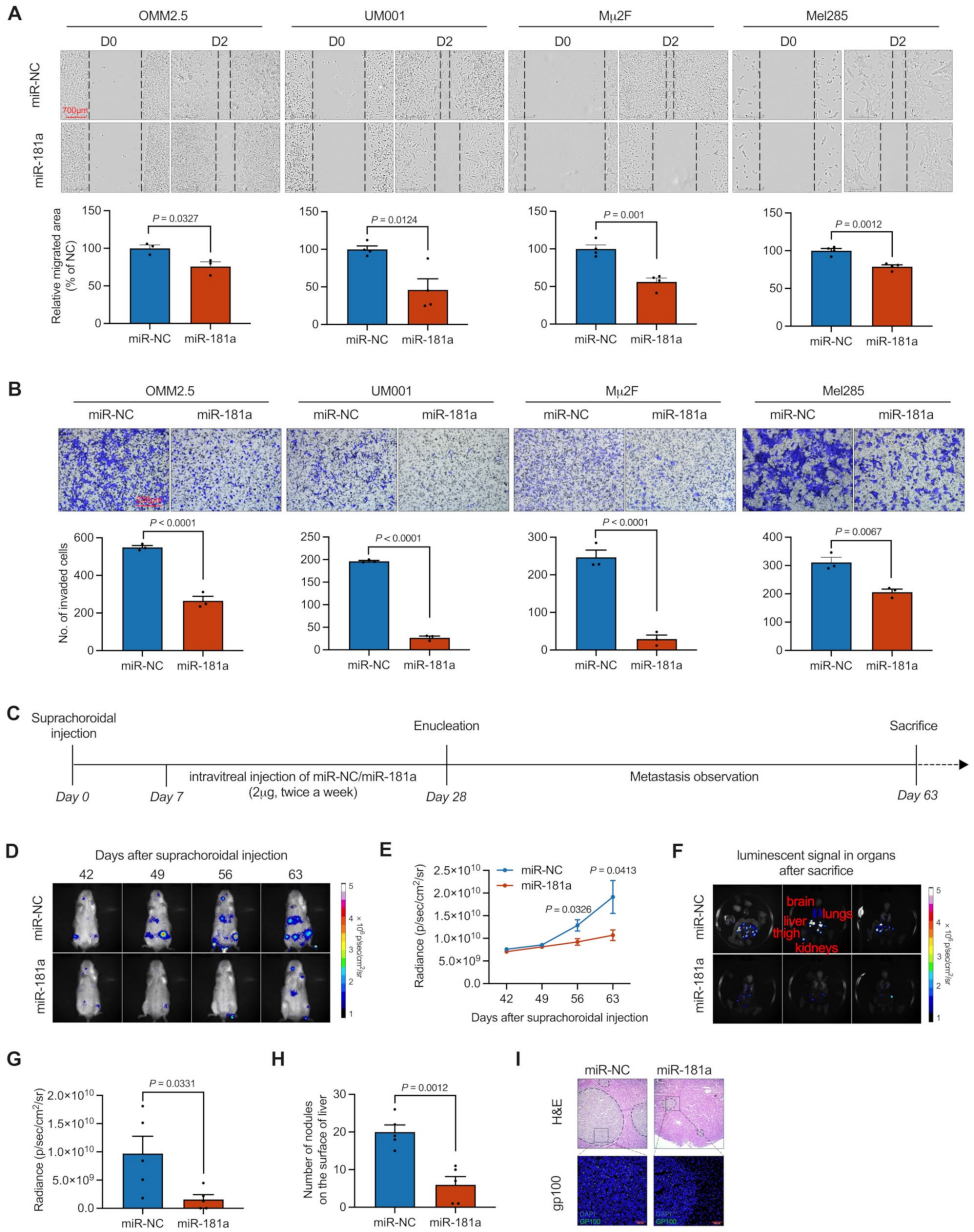
MiR-181a restrains metastasis in UM. (**A**) Photomicrograph (top) and quantitative analysis (bottom) of scratch wound healing assays from miR-NC and miR-181a (20 nM)-treated UM cells are shown. Scale bar: 700 μm. Data represent mean ± SEM (*n* = 3). (**B**) Forty-eight hours after incubation with 20 µM miR-181a, viable cells were counted and underwent Matrigel invasion assays. Representative images (top) for UM cells and quantitative analysis (bottom) from three random microscopic fields are shown. Data represent mean ± SEM. Scale bar: 200 μm. Data represent mean ± SEM (*n* = 3). (**C**) After suprachoroidal injection of 2 × 10⁶ OMM2.5-Luc cells, tumors were allowed to grow for 1 week. Subsequently, luminescence signal-positive NSG mice received intravitreal administration of either miR-NC or miR-181a (2 µg) twice a week for three weeks. After enucleation, metastases were observed. In vivo bioluminescence imaging was performed weekly for 8 weeks after suprachoroidal injection. (**D**) Representative images of luciferase signals from the entire body of animals in the miR-NC and miR-181a groups, taken from days 42 to day 63 after suprachoroidal injection, are shown. (**E**) Quantification of photon flux for metastases in NSG mice was performed every week after enucleation. Data represent mean ± SEM (*n* = 5). (**F**) Representative images of luciferase signals in metastatic organs after sacrifice are shown. (**G**) Quantification of photon flux from metastases in organs (brain, lungs, liver, kidneys, thigh bone) was performed after sacrifice. Data represent mean ± SEM (*n* = 5). (**H**) Surface metastatic nodules in the livers from each group were counted, and data are presented as mean ± SEM (*n* = 5). (**I**) Representative images of H&E staining and gp100 (UM marker) immunostaining in liver tissue sections are shown. Scale bar: 100 μm. *P* value was determined with two-tailed Student’s *t* test

We first analyzed the correlation between the level of miR-181a and UM patient prognosis by using TCGA and GEO datasets. Survival curves indicated that miR-181a level negatively correlated with both overall survival (Fig. S1A) and metastasis-free survival (Fig. S1B). We then evaluated the effects of miR-181a on the migration and invasion of four UM cell lines with distinct phenotypes in vitro. As shown in Fig. 1A, scratch wound healing assays of OMM2.5, UM001, Mµ2F, and Mel285 cells indicated a significant decrease in cell migration in response to miR-181a treatment. Analogously, the invasiveness of UM cells was considerably declined in the miR-181a-treated group, as assessed through Matrigel-coated Transwell chamber assays (Fig. 1B). Together, these findings revealed that miR-181a markedly suppresses the migration and invasion of UM cells.

We next used a metastasis model based on suprachoroidal injection of OMM2.5-Luc cells in NSG mice (Fig. S2A and B) to evaluate the effect of miR-181a on UM metastasis in vivo (Fig. 1C). The results indicated that intravitreal injection of miR-181a decreased the growth of suprachoroidal tumors (Fig. S2C). Metastases were detected on day 42 after suprachoroidal injection. Mice treated with miR-181a showed marked bioluminescence signal retardation during the development of metastasis in both the whole body (Fig. 1D and E) and organs collected after sacrifice (Fig. 1F and G). Because most advanced stage UM patients with metastasis develop liver metastasis, we dissected the liver and observed decreased numbers of metastatic tumor nodules on the liver surface after miR-181a treatment (Fig. 1H). Consistently, H&E staining and melanoma gp100 immunostaining analysis also indicated substantially smaller and fewer metastatic foci in the livers of miR-181a-treated mice than miR-NC-treated mice (Fig. 1I). These experimental results supported that miR-181a markedly abrogates UM metastasis in vivo.

### MiR-181a suppresses EMT and decreases cancer stem-like cells in UM

**Fig. 2:**
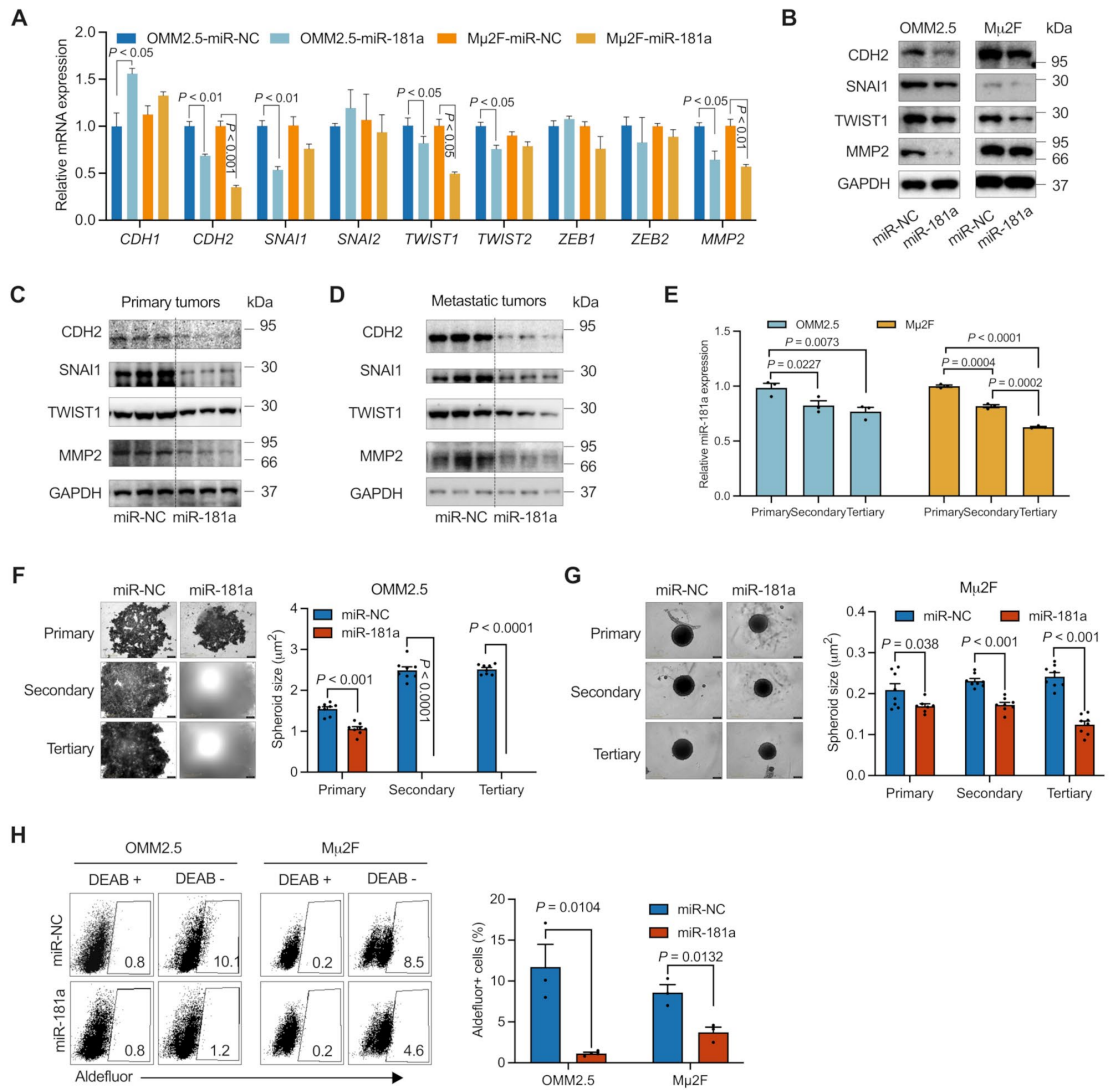
MiR-181a inhibits EMT and cancer stem cell characteristics. (**A** and **B**) Analyses of mRNA (**A**) and protein (**B**) expression of EMT markers in OMM2.5 and Mµ2F cells treated with 20 nM miR-NC or miR-181a for 48 h (mean ± SEM, *n* = 3) are shown. (**C** and **D**) Protein expression of EMT markers in primary (**C**) or liver metastatic (**D**) tumors collected from mice injected with OMM2.5-Luc cells and treated with 2 µg miR-NC or miR-181a (twice per week for three weeks) is shown. (**E**) OMM2.5 and Mµ2F cells were resuspended in melanosphere culture medium, seeded, and re-plated into ultra-low attachment 96-well plates for three rounds. The miR-181a levels in each round were measured with qRT-PCR (mean ± SEM, *n* = 3). (**F** and **G**) Forty-eight hours after treatment with 20 nM miR-NC or miR-181a, OMM2.5 (**F**) and Mµ2F (**G**) cells were resuspended in melanosphere culture medium, seeded, and re-plated into ultra-low attachment 96-well plates for three rounds. Left: representative images of melanospheres (scale bar: 400 μm). Right: quantification of melanosphere sizes. Data are presented as mean ± SEM (*n* = 8). (**H**) OMM2.5 and Mµ2F cells were incubated with 20 nM miR-NC or miR-181a for 48 h, and Aldefluor⁺ cells were measured with FACS, with or without the addition of an ALDH enzyme inhibitor (DEAB). Representative flow cytometry dot plots (left) for UM cells and quantitative analysis (right) from three independent experiments are shown. Data represent mean ± SEM (*n* = 3). *P* value was determined with two-tailed Student’s *t* test

EMT leads to a loss of epithelial cell polarity and epithelial markers, thereby enhancing cell motility. This process also promotes the maintenance of stem cell-like properties. Together, EMT and CSCs contribute to cancer metastasis [7]. To investigate the role of miR-181a in oncogenic transformation, we transfected OMM2.5 and Mµ2F cells with miR-181a. An increase in the epithelial marker E-cadherin (encoded by the *CDH1* gene) was observed in OMM2.5 cells (Fig. 2A). Notably, mesenchymal markers such as N-cadherin (encoded by *CDH2*), along with EMT transcription factors including *SNAI1* and *TWIST1*, were downregulated in both OMM2.5 and Mµ2F cells, as determined with quantitative reverse transcription polymerase chain reaction (qRT-PCR) assays (Fig. 2A), thus suggesting that miR-181a suppresses the EMT phenotype. Additionally, the expression of matrix metalloproteinase 2 (MMP2), a critical metastasis-associated protein [29], decreased in UM cells. The miR-181a-induced changes in mRNA levels of EMT markers were further validated at the protein level in both OMM2.5 and Mµ2F cells (Fig. 2B). Consistently, miR-181a decreased the expression of EMT markers in both primary and metastatic tumors harvested from mice (Fig. 2C and D). Collectively, these findings revealed that miR-181a decreases EMT in UM.

Given that miR-181a has been identified as a selective inhibitor of CSCs in multiple cancers [30, 31], we next investigated whether miR-181a might effectively target CSCs in UM. To assess self-renewal capability, we performed melanosphere formation and serial replating assays. The miR-181a levels decreased with each successive generation of melanospheres (Fig. 2E), and the melanosphere area size was significantly diminished in miR-181a-treated UM cells (Fig. 2F and G). Furthermore, miR-181a treatment markedly decreased the percentage of Aldefluor-positive cells in OMM2.5 and Mµ2F cells (Fig. 2H). These findings indicated that miR-181a exerts inhibitory effects on CSCs in UM.

### MiR-181a controls cholesterol biosynthesis in UM

**Fig. 3:**
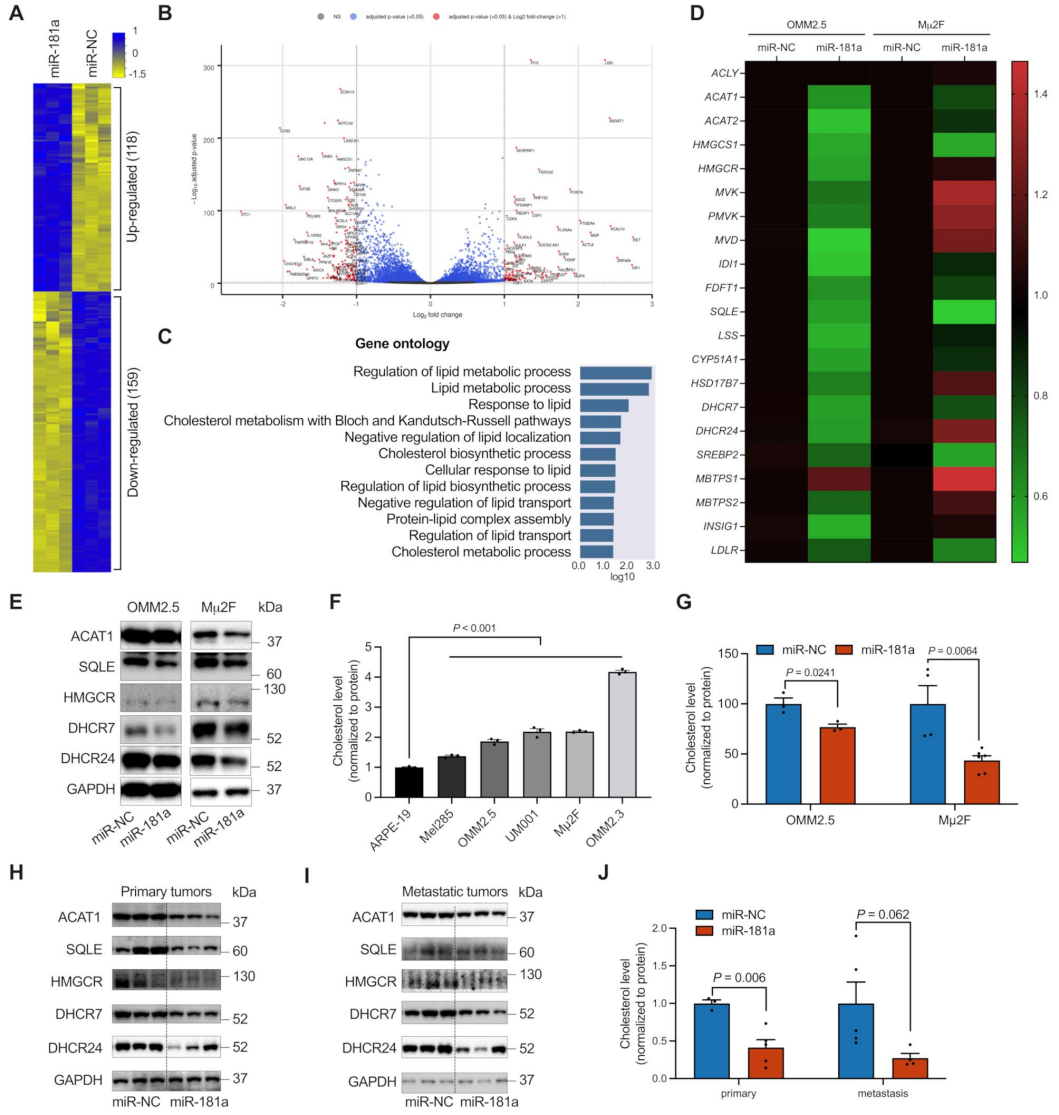
MiR-181a decreases cholesterol accumulation in UM. (**A** and **B**) Heatmap (**A**) and volcano plot (**B**) showing dysregulated genes identified by RNA-seq in OMM2.5 cells treated with 20 nM miR-NC or miR-181a. Data represent *n* = 3 biological replicates. (**C**) Gene Ontology (GO) enrichment analysis of miR-181a-induced down-regulated genes highlights their strong association with lipid metabolism. This analysis reveals key biological processes, molecular functions, and cellular components involved in lipid metabolic regulation, and offers insights into the mechanisms through which miR-181a modulates lipid metabolism. (**D**) RT-qPCR analysis of mRNA expression of cholesterol metabolism genes in OMM2.5 and Mµ2F cells treated with 20 nM miR-NC or miR-181a for 48 h. (**E**) Western blotting analysis of proteins involved in cholesterol metabolism in OMM2.5 and Mµ2F cells after treatment with 20 nM miR-NC or miR-181a for 48 h is shown. (**F**) Cholesterol levels in ARPE-19 and UM cell lines without treatment were measured with a cholesterol assay kit and normalized to protein levels (mean ± SEM, *n* = 3). (**G**) Cholesterol levels in OMM2.5 and Mµ2F cells treated with 20 nM miR-NC or miR-181a for 48 h were measured with a cholesterol assay kit and normalized to protein levels (mean ± SEM, *n* = 3). (**H** and **I**) Expression of cholesterol biosynthesis-associated proteins in primary (**H**) and metastatic (**I**) tumors collected from mice treated with 2 µg miR-NC or miR-181a (twice per week for 3 weeks) is shown. (**J**) Cholesterol levels in primary and metastatic tumors collected from mice treated with 2 µg miR-NC or miR-181a (twice per week for 3 weeks) were measured with a cholesterol assay kit and normalized to protein levels (mean ± SEM, *n* = 5). *P* value was determined with two-tailed Student’s *t* test

To elucidate the molecular mechanisms through which miR-181a decreases UM tumor metastasis, we conducted RNA sequencing (RNA-seq) analyses to compare the whole transcriptomes of OMM2.5 cells treated with miR-NC or miR-181a. In the miR-181a-treated group, 118 genes were upregulated, whereas 159 genes were downregulated (false discovery rate < 0.05 and fold change > 2; Fig. 3A and B). Gene ontology analysis revealed significant suppression of genes involved in cholesterol biosynthetic pathways in the miR-181a group (Fig. 3C). To validate the RNA-seq results, we performed qRT-PCR in both OMM2.5 and Mµ2F metastatic cells under the same experimental conditions. The mRNA expression of nearly all cholesterol biosynthesis genes, including *ACLY*, *ACAT1*, *ACAT2*, *HMGCS1*, *HMGCR*, *MVK*, *PMVK*, *MVD*, *IDI1*, *FDFT1*, *SQLE*, *LSS*, *CYP51A1*, *HSD17B7*, *DHCR7*, and *DHCR24*, was significantly downregulated in cells treated with miR-181a versus miR-NC (Fig. 3D). Additionally, miR-181a decreased the mRNA expression of key regulators of cholesterol biosynthesis, including *SREBF2*, *MBTPS1*, *MBTPS2*, and *INSIG1*, in UM cells (Fig. 3D). Furthermore, miR-181a decreased the expression of *LDLR*, the primary receptor of cholesterol influx from blood (Fig. 3D). In line with mRNA regulation, the levels of the key cholesterol biosynthesis related proteins ACAT1, SQLE, HMGCR, DHCR7, and DHCR24 decreased after miR-181a treatment (Fig. 3E). Collectively, these results suggested that miR-181a decreases levels of enzymes and transcriptional regulators of cholesterol biosynthesis.

Next, we assessed the cholesterol levels to determine whether miR-181a might ultimately regulate total cholesterol, given that UM cells exhibited higher total cholesterol levels than ARPE-19 cells (Fig. 3F). Total cholesterol levels significantly decreased in OMM2.5 and Mµ2F cells after miR-181a treatment (Fig. 3G). Additionally, we observed a marked decrease in cholesterol biosynthesis-associated proteins in both miR-181a-treated primary and metastatic tumors (Fig. 3H and I); correspondingly, total cholesterol levels also decreased in primary and metastatic tumors collected from mice (Fig. 3J). By integrating the expression data for cholesterol-regulating genes with cholesterol metabolic profiling, we concluded that miR-181a inhibits cholesterol biosynthesis and consequently decreases cholesterol accumulation in UM.

### Cholesterol restores miR-181a-decreased migration, invasion, EMT, and CSC characteristics in UM cells

**Fig. 4:**
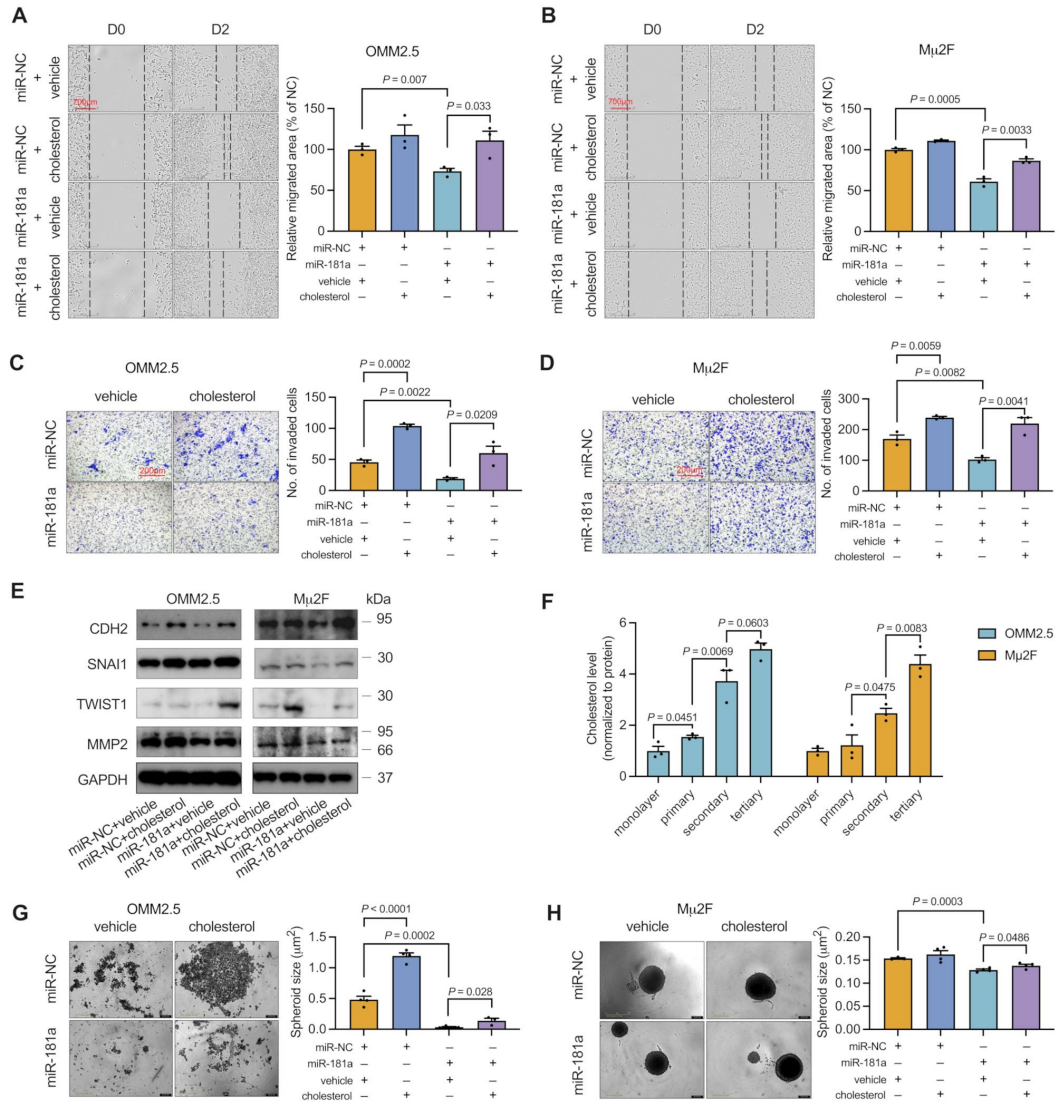
Cholesterol rescues the anti-metastatic effect of miR-181a. (**A** and **B**) Photomicrographs (left) and quantitative analyses (right) of the scratch wound healing assays in OMM2.5 (**A**) and Mµ2F (**B**) cells treated with miR-NC, cholesterol, miR-181a, or a combination of miR-181a and cholesterol are shown. Scale bar: 700 μm. Data represent mean ± SEM (*n* = 3). (**C** and **D**) Forty-eight hours after incubation with miR-NC, cholesterol, miR-181a, or miR-181a combined with cholesterol, viable cells were counted and subjected to Matrigel invasion assays. Representative images (left) for UM cells and quantitative analyses (right) from three random microscopic fields are shown. Scale bar: 200 μm. Data represent mean ± SEM (*n* = 3). (**E**) Protein expression of EMT markers in OMM2.5 and Mµ2F cells treated with miR-NC, cholesterol, miR-181a or miR-181a combined with cholesterol for 48 h is shown. (**F**) Untreated OMM2.5 and Mµ2F cells were resuspended in melanosphere culture medium, seeded, and re-plated into ultra-low attachment 96-well plates for three rounds. Cholesterol levels in monolayer cells and each round of spheroids were measured with a cholesterol assay kit and normalized to protein levels (mean ± SEM, *n* = 3). (**G** and **H**) Forty-eight hours after treatment with miR-NC, cholesterol, miR-181a, or miR-181a combined with cholesterol, OMM2.5 (**G**) and Mµ2F (**H**) cells were resuspended in melanosphere culture medium and seeded into ultralow-attachment 96-well plates. Left: representative images of melanospheres (scale bar: 400 μm). Right: quantification of melanosphere sizes. Data are presented as mean ± SEM (*n* = 4). *P* value was determined with one-way ANOVA corrected with Tukey’s test

To investigate the role of cholesterol in the miR-181a-induced inhibition of UM metastasis, we incubated OMM2.5 and Mµ2F cells with water-soluble cholesterol (5 µg/mL) after miR-181a transfection. Cholesterol successfully promoted migration and invasion of UM cells and rescued the diminished migration (Fig. 4A and B) and invasion (Fig. 4C and D) abilities of UM cells treated with miR-181a. In agreement with these findings, cholesterol significantly reversed the EMT inhibition mediated by miR-181a (Fig. 4E). We then evaluated whether cholesterol might be involved in the miR-181a-induced inhibition of CSCs. Cholesterol levels were progressively elevated with increasing generation of cells, as compared with cells in monolayer (Fig. 4F). Similarly, cholesterol treatment negated the miR-181a-induced suppression of stemness (Fig. 4G and H). Together, these results indicated that cholesterol restores the miR-181a induced inhibition of migration, invasion, EMT, and maintenance of CSCs in UM.

### High SREBP2 expression correlates with poor prognosis of UM patients

**Fig. 5:**
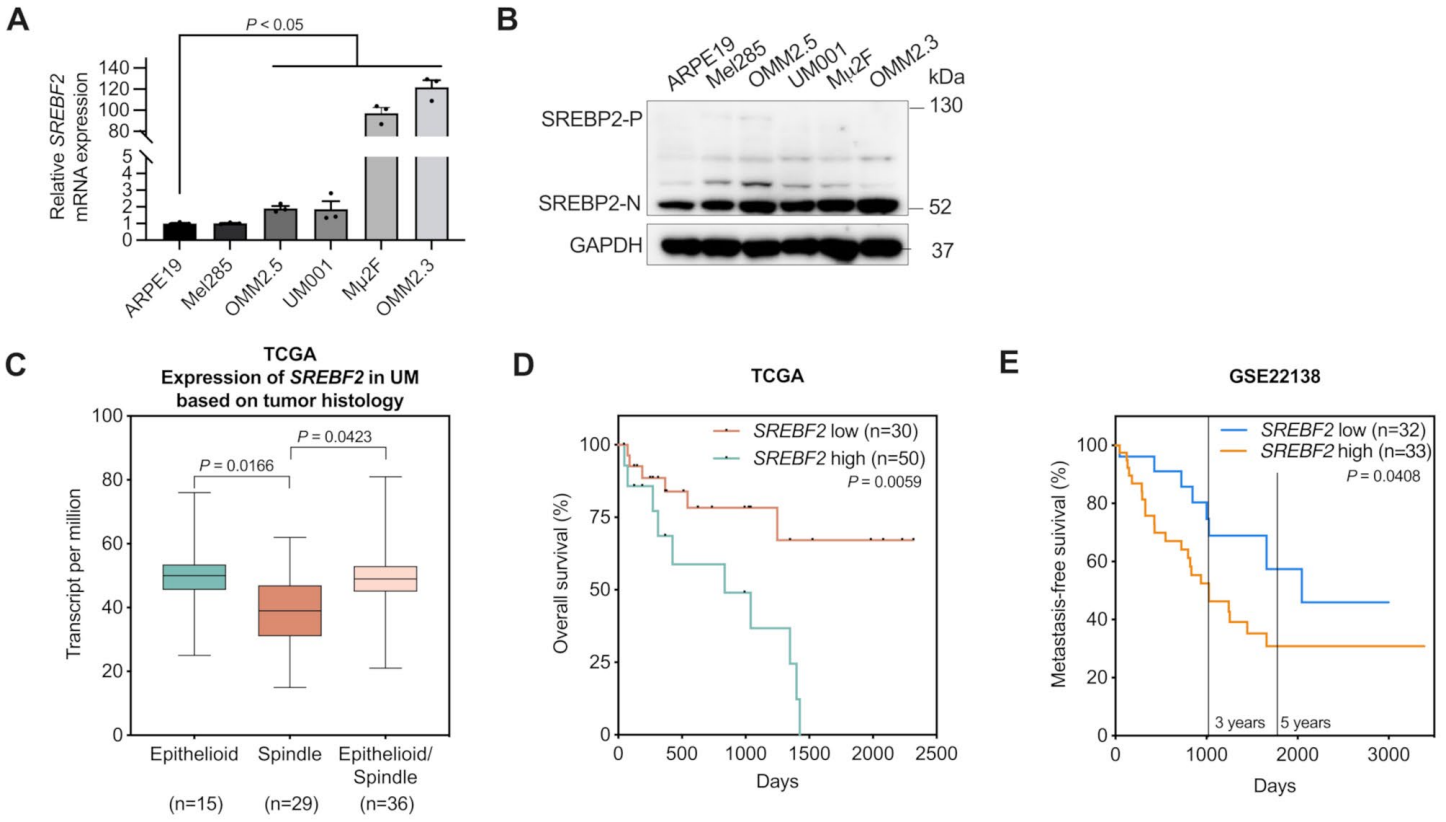
SREBP2 is overexpressed in UM. (**A**) The expression of the *SREBF2* mRNA in ARPE-19 and UM cells was detected by qRT-PCR. Data are shown as mean ± SEM (*n* = 3, one-way ANOVA, multiple comparisons, post hoc comparison by Tukey test). (**B**) Western blotting analysis of the expression of SREBP2 in ARPE-19 and UM cells is shown. (**C**) The expression of SREBP2 was analyzed in various cell types (spindle, epithelioid and mixed) of UM primary tumors (TCGA datasets, *n* = 80, Welch’s T-test). (**D**) High expression of the *SREBF2* mRNA was negatively correlated with overall survival in patients with UM (TCGA datasets, *n* = 80, log-rank test). (**E**) High expression of the *SREBF2* mRNA was negatively correlated with metastasis-free survival in patients with UM (GEO database, Series #GSE22138, *n* = 65 primary tumors, log-rank test)

SREBP2 (encoded by *SREBF2*), a key transcriptional regulator of cholesterol biosynthesis, specifically modulates genes encoding cholesterol-synthesizing enzymes [17]. Notably, *SREBF2* was identified as a potential target of miR-181a, according to miRNA databases. To explore the relationship between SREBP2 and UM, we first assessed the expression of SREBP2 in normal control ARPE-19 cells and UM cells through Western blotting and qRT-PCR. Despite the varying expression levels of SREBP2 among UM cell lines because of their distinct phenotypes, SREBP2, particularly its cleaved form (SREBP2-N), was overexpressed in UM cells at both the mRNA (Fig. 5A) and protein (Fig. 5B) levels. Additionally, higher levels of *SREBF2* mRNA were observed in epithelioid or mixed (epithelioid and spindle) cells than spindle cells (Fig. 5C). Epithelioid or mixed cell types may potentially predict poorer survival outcomes among patients with UM [32, 33]. We further analyzed the correlation between the *SREBF2* expression and UM patient prognosis by using TCGA (80 primary tumors from patients with UM) and GEO (65 patients with primary UM) datasets [34]. Survival curves indicated that *SREBF2* expression negatively correlated with both overall survival (Fig. 5D) and metastasis-free survival (Fig. 5E); therefore, SREBP2 might have a potential role in the metastatic progression of UM.

### MiR-181a suppresses the cholesterol biosynthesis and UM metastasis by targeting SREBP2

**Fig. 6:**
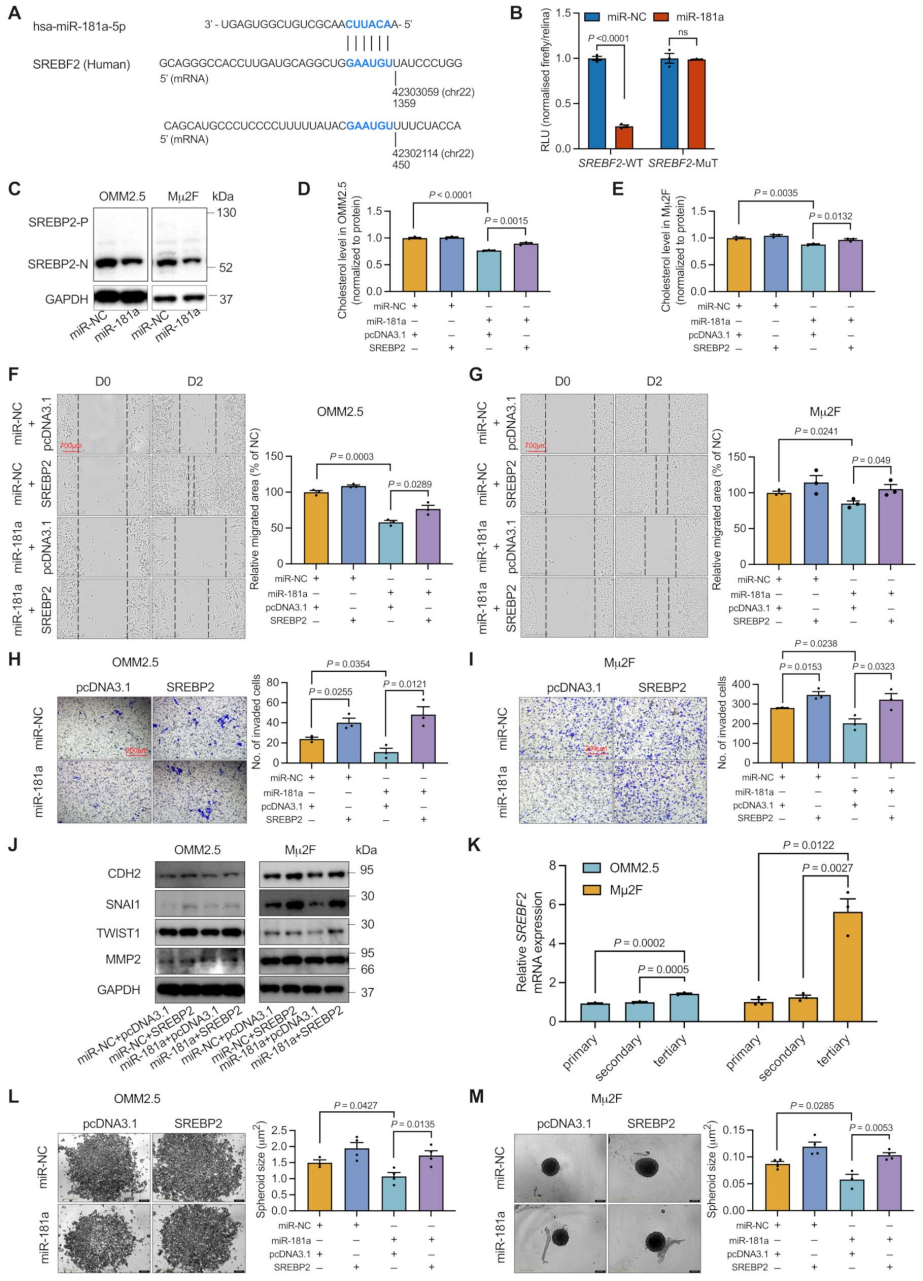
MiR-181a inhibits metastasis by targeting the ***SREBF2*** mRNA. (**A**) Diagram of the two locations and the alignments of miR-181a to the 3’UTR of the human SREBF2 transcript is shown. (**B**) A pmirGLO vector containing a wild-type or mutated 3’UTR of the SREBP2 mRNA was transfected with miR-181a into 293T cells. Firefly luciferase activity, in relative light units (RLU), was analyzed 48 h after transfection and normalized to that of *Renilla* luciferase (pRL-TK). Reporter activities of cells transfected with miR-NC were used as avcontrol. Data are presented as mean ± SEM (*n* = 3). (**C**) Western blotting analysis of SREBP2 levels in OMM2.5 and Mµ2F cells after treatment with 20 nM miR-NC or miR-181a for 48 h is shown. (**D** and **E**) Cholesterol levels in OMM2.5 (**D**) and Mµ2F (**E**) cells treated with miR-NC, SREBP2, miR-181a or miR-181a and SREBP2 for 48 h were measured with a cholesterol assay kit and normalized to protein levels (mean ± SEM, *n* = 3). (**F** and **G**) Photomicrographs (left) and quantitative analyses (right) of scratch wound healing assays in OMM2.5 (**F**) and Mµ2F (**G**) cells treated with miR-NC, SREBP2, miR-181a, or a combination of miR-181a and SREBP2 are shown. Scale bar: 700 μm. Data represent mean ± SEM (*n* = 3). (**H** and **I**) Forty-eight hours after incubation with miR-NC, SREBP2, miR-181a, or miR-181a combined with SREBP2, viable OMM2.5 (**H**) and Mµ2F (**I**) cells were counted and subjected to Matrigel invasion assays. Representative images (left) for UM cells and quantitative analyses (right) from three random microscopic fields are shown. Scale bar: 200 μm. Data represent mean ± SEM (*n* = 3). (**J**) Protein expression of EMT markers in OMM2.5 and Mµ2F cells treated with miR-NC, SREBP2, miR-181a, or miR-181a combined with SREBP2 for 48 h (mean ± SEM, *n* = 3). (**K**) Untreated OMM2.5 and Mµ2F cells were resuspended in melanosphere culture medium, seeded, and re-plated into ultralow-attachment 96-well plates for three rounds. *SREBF2* levels in each round of spheroids were measured by RT-qPCR (mean ± SEM, *n* = 3). (**L** and **M**) Forty-eight hours after treatment with miR-NC, SREBP2, miR-181a, or miR-181a combined with *SREBF2*, OMM2.5 (**L**) and Mµ2F (**M**) cells were resuspended in melanosphere culture medium and seeded into ultra-low attachment 96-well plates. Left: representative images of melanospheres (scale bar: 400 μm). Right: quantification of melanosphere sizes. Data are presented as mean ± SEM (*n* = 4). *P* value was determined with two-tailed Student’s *t* test (**B**) and one-way ANOVA corrected with Tukey’s test (**D-I** and **K-M**)

According to information from the miRNA database, we identified a conserved sequence in the 3’UTR of the *SREBF2* mRNA that matches the seed region for miR-181a (Fig. 6A). Through dual-luciferase assays and transient transfection of miR-181a, we confirmed that miR-181a indeed decreased *SREBF2* expression, and deletion of the micro-RNA-binding site within the 3’UTR of *SREBF2* mRNA abolished this regulation (Fig. 6B). We then created a miR-181a overexpression in UM cell lines and observed that elevated miR-181a levels significantly decreased cleaved SREBP2 (SREBP2-N) expression in OMM2.5 and Mµ2F cells (Fig. 6C). These findings indicated that miR-181a directly targets SREBP2 in UM cells.

To further explore the role of SREBP2 in cholesterol biosynthesis, we used both SREBF2 silencing and overexpression in UM cells, then detected SREBP2 levels after treatment (Fig. S3A and B). Silencing of SREBP2 with siRNA (siR-SREBP2) decreased the total cholesterol levels in OMM2.5 and Mµ2F cells (Fig. S3C), whereas overexpression of SREBP2 counteracted the decrease in cholesterol levels induced by miR-181a (Fig. 6D and E).

To assess the involvement of SREBP2 in the miR-181a-induced inhibition of UM metastasis, we treated OMM2.5 and Mµ2F cells with SREBP2 silencing or overexpression after miR-181a transfection. Silencing of SREBP2 significantly decreased the migration ability of UM cells (Fig. S3D and E). However, SREBP2 overexpression effectively promoted the migration and invasion of UM cells and restored the effects on cell migration (Fig. 6F and G) and invasion (Fig. 6H and I) elicited by miR-181a. In agreement with these findings, SREBP2 significantly reversed the EMT inhibition caused by miR-181a (Fig. 6J). We also examined whether SREBP2 might be implicated in the miR-181a-induced suppression of CSCs. *SREBF2* mRNA levels increased with higher cell generations, as compared with cells in monolayer (Fig. 6K). Similarly, SREBP2 overexpression reversed the miR-181a-induced decrease in stemness (Fig. 6L and M). Given the critical role of the AKT signaling pathway in lipid metabolism and our previous findings indicating that miR-181a regulates AKT3 signaling, we investigated the potential crosstalk between the AKT3 and SREBP2 pathways. Western blot analysis provided direct evidence supporting a regulatory relationship between AKT3 and SREBP2 in UM, specifically demonstrating that miR-181a-mediated modulation of AKT3 plays a significant role in the regulation of SREBP2 (Fig. S3F).

Collectively, these results demonstrated that miR-181a targets *SREBF2*, and consequently inhibits cholesterol biosynthesis, migration, invasion, EMT, and maintenance of CSCs in UM.

### Combinational treatment with miR-181a and Crizotinib prevents UM metastasis

**Fig. 7:**
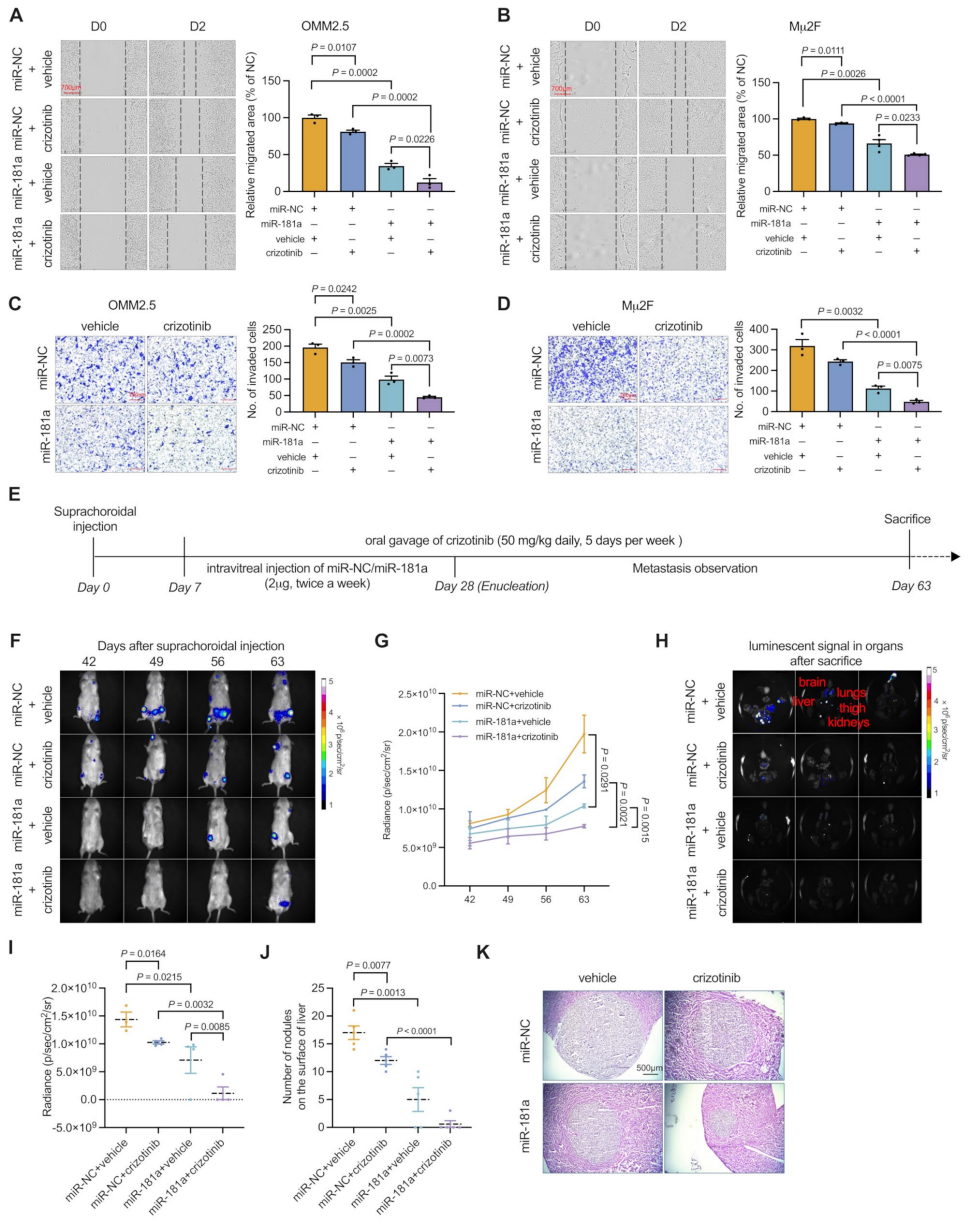
Combination treatment with miR-181a and crizotinib prevents metastasis in UM. (**A** and **B**) Photomicrographs (left) and quantitative analyses (right) of scratch wound healing assays in OMM2.5 (**A**) and Mµ2F (**B**) cells treated with miR-NC, crizotinib, miR-181a, or a combination of miR-181a and crizotinib are shown. Scale bar: 700 μm. Data represent mean ± SEM (*n* = 3). (**C** and **D**) Forty-eight hours after incubation with miR-NC, crizotinib, miR-181a, or miR-181a combined with crizotinib, viable OMM2.5 (**C**) and Mµ2F (**D**) cells were counted and subjected to Matrigel invasion assays. Representative images (left) for UM cells and quantitative analyses (right) from three random microscopic fields are shown. Scale bar: 200 μm. Data represent mean ± SEM (*n* = 3). (**E**) After suprachoroidal injection of 2 × 10⁶ OMM2.5-Luc cells, tumors were allowed to grow for 1 week. Subsequently, luminescence signal-positive NSG mice received intravitreal administration of either miR-NC or miR-181a (2 µg) twice per week for 3 weeks and vehicle or crizotinib (50 mg/kg daily) 5 days per week for 8 weeks. After enucleation, metastases were observed. In vivo bioluminescence imaging was performed weekly for 8 weeks after suprachoroidal injection. (**F**) Representative images of luciferase signals from the entire body in animals in the groups receiving miR-NC, crizotinib, miR-181a, or a combination of miR-181a and crizotinib, taken from days 42 to 63 after suprachoroidal injection, are shown. (**G**) Quantification of photon flux for metastases in NSG mice was performed every week after enucleation. Data represent mean ± SEM (*n* = 5). (**H**) Representative images of luciferase signals in metastatic organs (brain, lungs, liver, kidneys, thigh bone) after sacrifice are shown. (**I**) Quantification of photon flux from metastases in organs was performed after sacrifice. Data represent mean ± SEM (*n* = 5). (**J**) Surface metastatic nodules in the liver from each group were counted, and data are presented as mean ± SEM (*n* = 5). (**K**) Representative images of H&E staining in liver tissue sections are shown. Scale bar: 500 μm. *P* value was determined with one-way ANOVA corrected with Tukey’s test

Crizotinib, a selective small-molecule inhibitor targeting c-Met, has been demonstrated to reduce UM metastasis [22]. We confirmed that crizotinib inhibited migration ability in a dose-dependent manner (Fig. S4A and B). To enhance the survival rate of patients with metastatic UM, we explored the combined effects of miR-181a and crizotinib on UM cells and in a murine metastatic UM model. We first evaluated the effects of combined miR-181a and crizotinib treatment on the migration and invasion of UM cells in vitro. As demonstrated in Fig. 7A and B, scratch wound healing assays with OMM2.5 and Mµ2F cells showed significantly less migration after combination treatment than after treatment with either miR-181a or crizotinib alone. Similarly, the invasiveness of UM cells was markedly diminished in the combination treatment group, as determined with the Matrigel-coated Transwell chamber assays (Fig. 7C and D). Collectively, these results indicated that the combined use of miR-181a and crizotinib substantially suppresses migration and invasion in UM cells. In addition, when combined with miR-181a (25 nM), crizotinib exhibited an additive inhibitory effect on UM cell migration. The inhibitory rate of the combination closely approximates the sum of miR-181a alone and crizotinib alone. Similar additive effects were observed across a range of crizotinib concentrations (Fig. S5 A and B).

To evaluate the combinatory effects of miR-181a and crizotinib on UM metastasis in vivo, we also used a metastasis model based on suprachoroidal injection of OMM2.5-Luc cells in NSG mice (Fig. 7E). The findings demonstrated a significant decrease in bioluminescence signals in mice treated with miR-181a and crizotinib during the development of metastasis, as observed both in whole-body imaging (Fig. 7F and G) and in colleted organs after sacrifice (Fig. 7H and I). These finding were accompanied by a decrease in the numbers of metastatic tumor nodules on the liver surface (Fig. 7J). In agreement with these observations, histological analyses, including H&E staining and gp100 immunostaining, revealed markedly smaller and fewer metastatic foci in the livers of mice subjected to combination therapy than to treatment with miR-181a or crizotinib alone (Fig. 7K). These results robustly indicated that the combined use of miR-181a and crizotinib effectively prevents UM cell metastasis in vivo.

## Discussion

Metastasis of UM remains an unresolved challenge. This study is the first to demonstrate that exogenous miR-181a can be utilized to treat UM metastasis by targeting SREBP2-mediated cholesterol biosynthesis, with EMT and CSC characteristics implicated in this process. The present study provides the first evidence that miR-181a effectively suppresses migration and invasion in vitro and significantly impedes metastasis in vivo. Our results indicated that miR-181a effectively inhibited UM cell migration regardless of whether the cell line originated from primary tumors or liver metastases of patients, thus highlighting its broad effects in UM. Previous research in our laboratory using a subcutaneous tumor model has revealed that miR-181a suppresses UM tumor growth through targeting the constitutive activated oncogenic PI3K/AKT pathway [6]. Although the decreased metastasis observed herein might have partially resulted from the suppression of primary tumor growth, our in vitro scratch and invasion assays controlled for cell number, thereby excluding the possibility that this variable affected the metastasis outcome. Importantly, the primary goal of using miR-181a, for preventing metastasis, was achieved through localized miR-181a injections. Therefore, miR-181a might serve as a promising therapeutic option for patients with UM to prevent metastatic spread.

A key strength of our study is the use of suprachoroidal injection in an animal model, which has greater clinical relevance than other metastasis models such as intrasplenic transplantation, tail vein, or intravitreal injection [28, 35, 36]. A previous study using an orthotopic mouse model of UM has reported metastases in the lungs, liver, and kidneys [37]. Our results identified metastases in the liver, brain, lymph nodes, lungs, kidneys, and bones, in agreement with previous findings [38].

Regarding the mechanism through which miR-181a inhibits metastasis, miR-181a exerts potent inhibitory effects on EMT and CSCs in UM and significantly decreases cholesterol biosynthesis. A prior study has reported that higher mRNA levels of EMT-associated genes are related with a more aggressive clinical phenotype in UM samples, and EMT-associated factors enhance the invasive properties of UM cells, suggesting these factors might serve as novel therapeutic targets in patients [39]. Additionally, the “seed” characteristics of CSCs have led to growing interest in their targeting for therapeutic intervention. Recent studies have demonstrated that inhibitors of CSCs can effectively decrease metastasis in various UM cell lines [28, 40–42]. Given that miR-181a suppresses EMT and CSCs in UM, as demonstrated in other cancers [43], it effectively prevents metastasis.

Previous studies have shown that elevated cholesterol levels promote metastasis in cancer [11–13] and our current research revealed that cholesterol levels play critical roles in the miR-181a-induced suppression of EMT and CSCs in UM. Cholesterol not only reverses the effects of miR-181a on EMT and CSCs but also ultimately restores its effects on metastasis. Moreover, our data suggested that SREBP2 contributes to the miR-181a-mediated elimination of EMT and CSCs, as well as the restriction of migration and invasion in UM. In the nucleus, SREBP2-N activates the expression of genes encoding key enzymes in the mevalonate pathway, such as HMGCR, MVK, SQS, and DHCR24, and also upregulates LDLR, thus facilitating exogenous cholesterol uptake [44]. By targeting SREBP2, miR-181a modulates multiple steps of cholesterol biosynthesis and transport, thereby exerting potent inhibitory effects on metastasis processes associated with cholesterol. Additionally, multiple key pathways, including the PI3K/AKT signaling pathway, activate SREBP-2 and promote tumorigenesis [44]. Our previous study has demonstrated that miR-181a directly targets AKT3 [6]; therefore, miR-181a may regulate cholesterol biosynthesis both directly and indirectly by targeting cholesterol biosynthesis-associated genes.

In recent years, combination therapies have been extensively studied, but their success has often been limited by substantial adverse effects or insufficient efficacy in eradicating tumor cells.

**Fig. 8:**
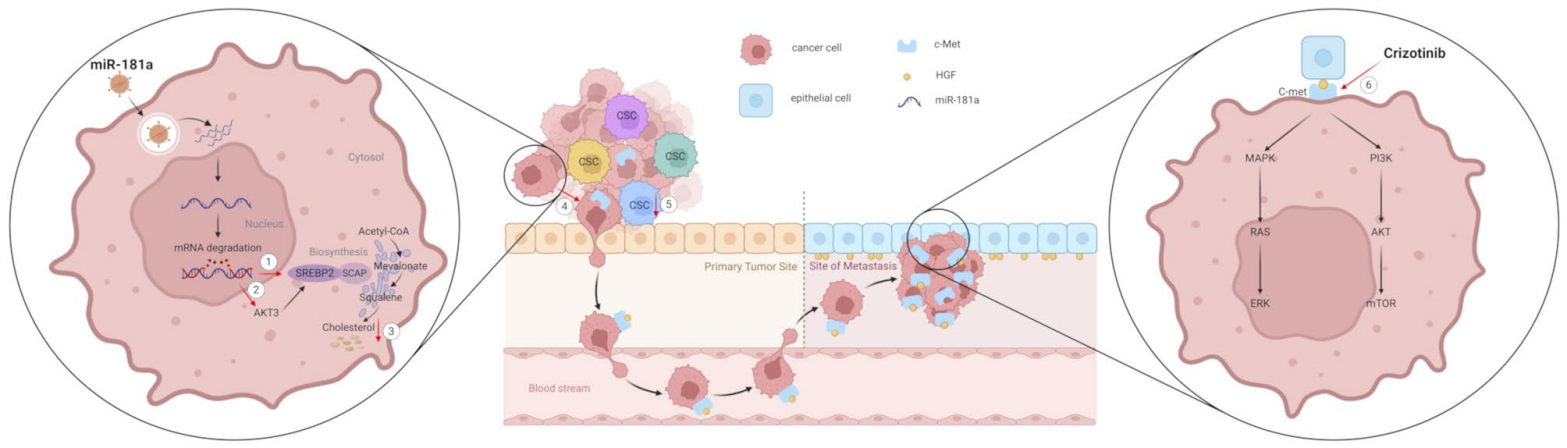
Overall schematic presentation of the combinatory effect of miR-181a and crizotinib in UM. After transfection, miR-181a downregulates the mRNA levels of *SREBF2* and *AKT3*. ① Downregulation of SREBP2 impairs promotion of cholesterol biosynthesis. ② Decreased levels of AKT3 not only limit the proliferation and migration of UM cells but also hinder the upregulation of cholesterol biosynthesis. ③ Together, these processes lead to lower cholesterol levels in UM cells. ④ Decreased cholesterol levels hinder EMT, thereby limiting the migration of UM cells from the primary tumor to the bloodstream. ⑤ Lower cholesterol levels also decrease the number of CSCs, thereby inhibiting colonization at metastatic sites. ⑥ Meanwhile, crizotinib inhibits c-Met activation, suppressing key downstream signaling pathways involved in cholesterol biosynthesis, cell survival, proliferation and migration. Overall, the combination of miR-181a and crizotinib effectively prevents UM metastasis by inhibiting both the migration of primary tumor cells and the colonization of tumor cells at metastatic sites

Crizotinib, an inhibitor of c-Met (the receptor for HGF), blocks the effects of HGF, a growth factor essential for the proliferation, migration, and survival of epithelial and endothelial cells involved in tissue repair across various organs, including the heart, lungs, kidneys, liver, brain, and bones [20]. Crizotinib was initially found to be effective in treating UM metastasis; however, its use as an adjuvant therapy in high-risk patients with UM did not improve recurrence-free survival, because crizotinib alone does not decrease the viability of UM cells [45]. These findings have led to the exploration of new drug combinations with crizotinib to better prevent metastasis. Crizotinib inhibits downstream signaling pathways of c-Met, such as MAPK/ERK and PI3K/AKT (Fig. 8), which are also inhibited by miR-181a, thus suggesting a potential synergistic effects in regulating cholesterol biosynthesis, survival, proliferation, and migration of UM cells. Our data indicated that combining miR-181a with crizotinib effectively prevented UM metastasis by not only inhibiting the migration of primary tumor cells but also preventing the colonization of tumor cells at metastatic sites. This combination may offer a promising strategy to prevent metastasis in patients with UM (Fig. 8). These studies also inspired us to further investigate the combined effect of miR-181a and crizotinib on the treatment of patients with metastatic UM.

In summary, miR-181a exhibits substantial inhibitory effects on UM metastasis both in vitro and in vivo. It decreases cholesterol levels in UM cells, at least in part, by repressing SREBP2. Additionally, miR-181a suppresses the EMT process and restricts CSCs in UM cells. Our findings elucidate the molecular mechanisms underlying the anti-metastatic activity of miR-181a and support the potential of a clinical trial exploring the combined use of miR-181a and crizotinib in patients with UM, to prevent and suppress metastasis.

## Conclusions

MiR-181a suppresses UM metastasis by downregulating SREBP2 and disrupting cholesterol biosynthesis. Its combination with crizotinib holds potential as an effective therapeutic approach for metastatic UM.

## Electronic supplementary material

Below is the link to the electronic supplementary material.

Supplementary Material 1

## Data Availability

No datasets were generated or analysed during the current study.

## Abbreviations

UM: Uveal melanoma
CSC: Cancer stem-like cell
SREBP2: Sterol regulatory element-binding protein 2
EMT: Epithelial-to-mesenchymal transition
CSC: Cancer stem-like cell
HGF: Hepatic growth factor
NSG: NOD.Cg-Prkdcscid Il2rgtm1Wjl/SzJ
SDS-PAGE: Sodium dodecyl sulfate polyacrylamide gel electrophoresis
TBST: Tris Buffered Saline with Tween 20
ALDH: Aldehyde dehydrogenase
NC: Mimic-negative control
RLU: Relative light units
RNA-seq: RNA sequencing
