# Supplementary material for: MiR-181a-driven downregulation of cholesterol biosynthesis through SREBP2 inhibition suppresses uveal melanoma metastasis
Source: J Exp Clin Cancer Res. 2025 Jul 19;44:215. doi: 10.1186/s13046-025-03459-8 (PMC12275384; doi:10.1186/s13046-025-03459-8)
Supplement: Supplementary file 1 — Supplementary Material 1 [file 13046_2025_3459_MOESM1_ESM.pdf]

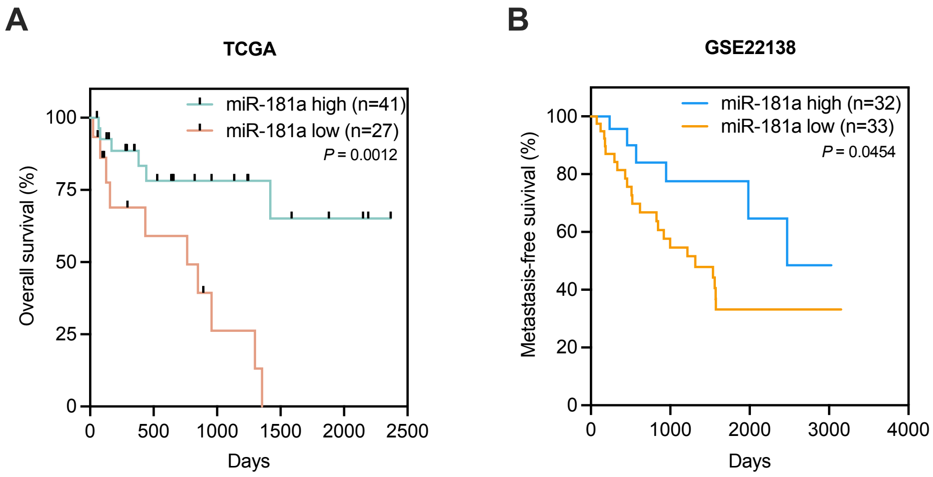

Supplementary Fig. 1. Low miR-181a level correlates with poor prognosis of UM patients. A High miR-181a level was positively correlated with overall survival in patients with UM (TCGA datasets, n=68, log-rank test). **B** High miR-181a level was positively correlated with metastasis-free survival in patients with UM (GEO database, Series #GSE22138, n=65 primary tumors, log-rank test)

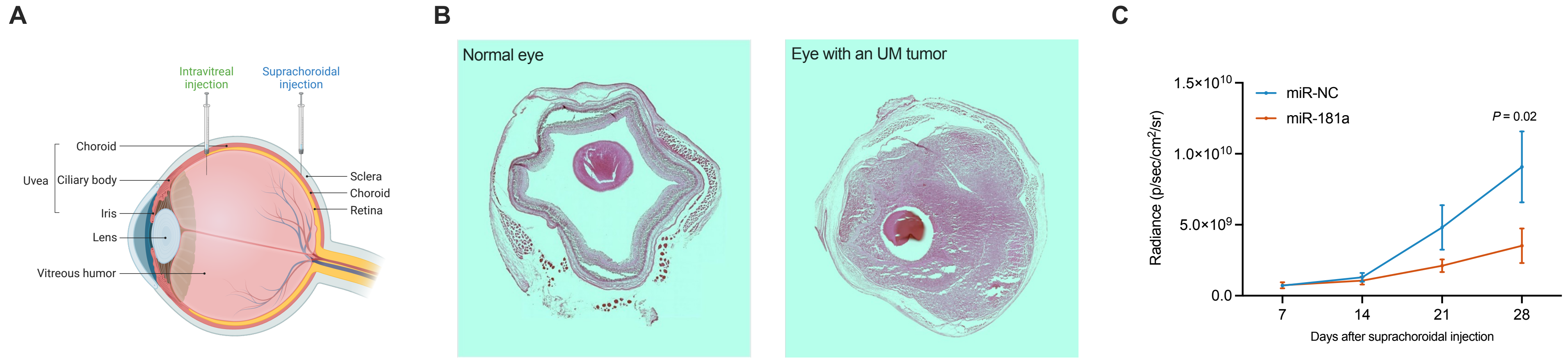

Supplementary Fig. 2. MiR-181a inhibits growth of suprachoroidal tumors. **A** Diagram of suprachoroidal Injection and intravitreal injection. **B** Twenty-eight days after the suprachoroidal injection, eyeballs with or without injection of UM cells were harvested. H&E staining revealed that the layers in the normal eye were defined, whereas the layers in the eye with the UM tumor were indistinct, and the entire vitreous cavity was occupied by the tumor. **C** Quantification of photon flux for primary tumors in NSG mice treated with miR-NC or miR-181a was performed every week after suprachoroidal injection of OMM2.5-Luc cells. Data represent mean ± SEM (n = 5). *P* value was determined with one-way ANOVA corrected with Tukey's test.

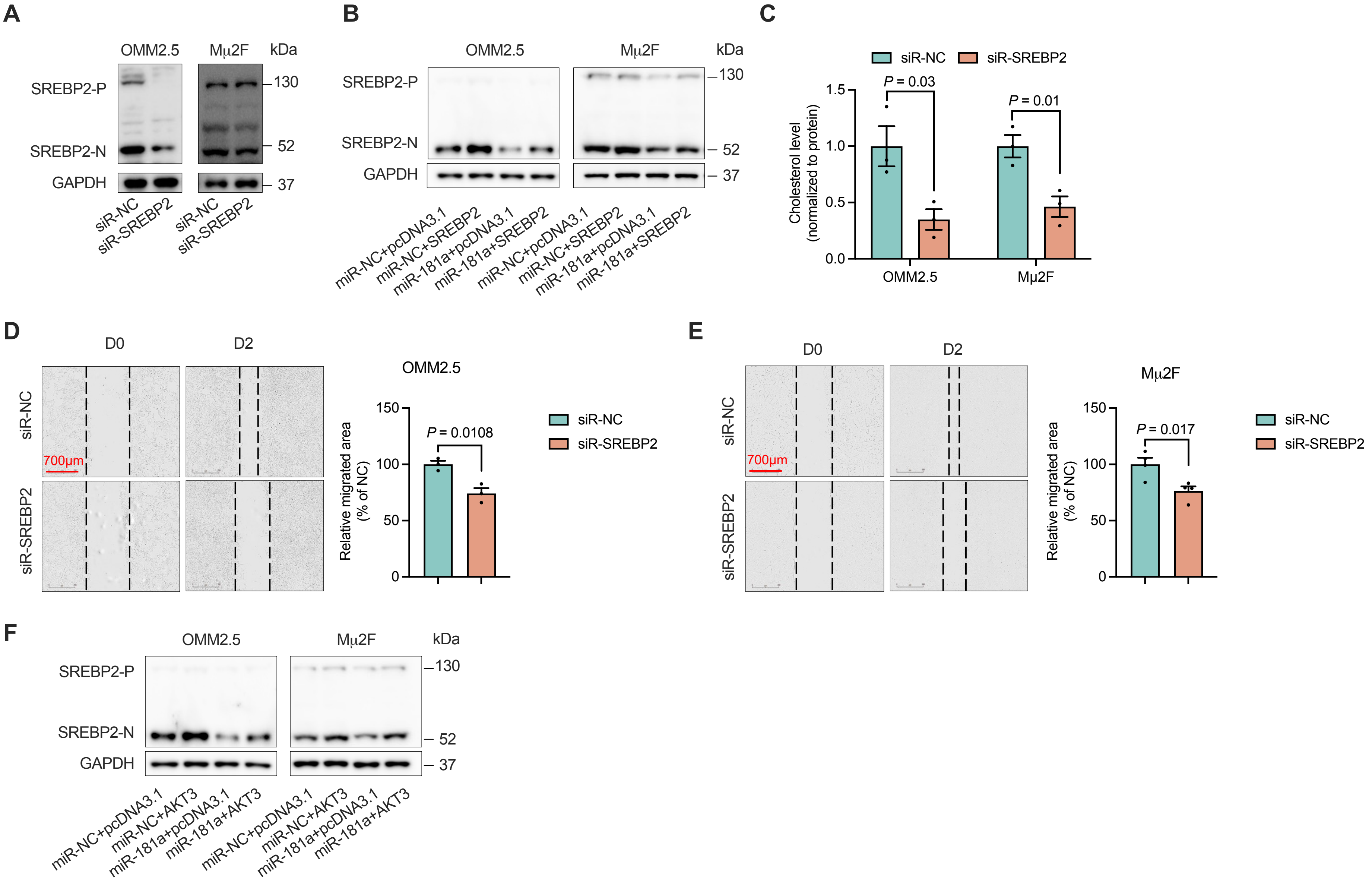

Supplementary Fig. 3. SREBP2 silencing decreases UM cell migration and cholesterol levels. A, **B** Western blotting analysis of SREBP2 levels in OMM2.5 and Mμ2F cells after treatment with SREBP2 silencing (**A**) or SREBP2 overexpression (**B**) for 48 hours is shown. **C** Cholesterol levels in OMM2.5 and Mμ2F cells treated with siR-NC or siR-SREBP2 for 48 hours were measured with a cholesterol assay kit and normalized to protein levels (mean ± SEM, n = 3). **D, E** Photomicrographs (left) and quantitative analyses (right) of the scratch wound healing assays in OMM2.5 (**D**) and Mμ2F (**E**) cells treated with siR-NC or siR-SREBP2 are shown. **F** Western blotting analysis of SREBP2 levels in OMM2.5 and Mμ2F cells after treatment with miR-181a or AKT3 overexpression for 48 hours is shown. Scale bar: 700 μm. Data represent mean ± SEM (n = 3). *P* value was determined with one-way ANOVA corrected with Tukey’s test.

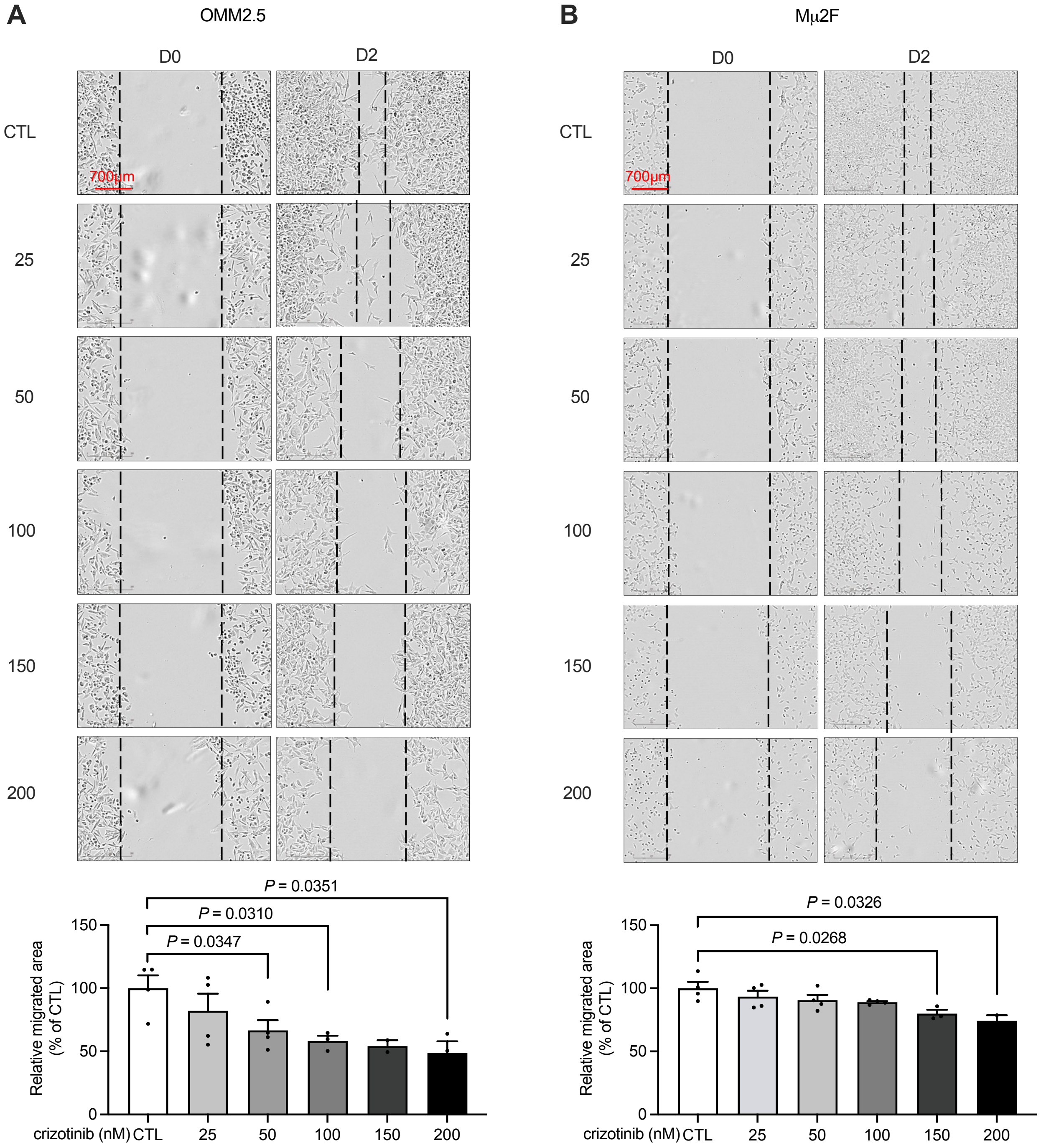

Supplementary Fig. 4. Crizotinib inhibits the migration ability of UM cells in a dose-dependent manner. Photomicrographs (top) and quantitative analyses (bottom) of the scratch wound healing assays in OMM2.5 (**A**) and Mμ2F (**B**) cells treated with crizotinib (25-200 nM) are shown. Scale bar: 700 μm. Data represent mean ± SEM (n = 4). *P* value was determined with one-way ANOVA corrected with Tukey's test.

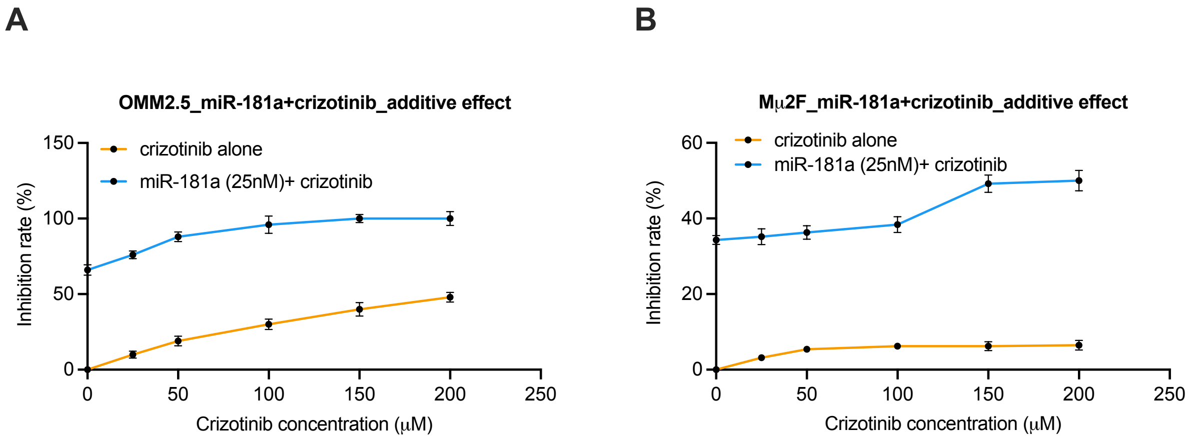

Supplementary Fig. 5. Additive inhibitory effect of miR-181a and crizotinib on UM cell migration and invasion. Scratching assay results showing the inhibition rates of OMM2.5 (**A**) and Mμ2F (**B**) cells under different concentrations of crizotinib alone or in combination with miR-181a (25 nM). The combination group exhibited inhibition rates approximately equal to the sum of the individual effects of miR-181a and crizotinib, indicating an additive effect.

Table S1. List of primers used for qRT-PCR analyses

Human mRNA transcript or vector	Forward primer sequence	Reverse primer sequence
ACLY	ATCGGTTCAAGTATGCTCGGG	GACCAAGTTTTCCACGACGTT
ACAT1	ATGCCAGTACACTGAATGATGG	GATGCAGCATATACAGGAGCAA
ACAT2	CTTTAGCACGGATAGTTTCCTGG	GCTGCAAAGGCTTCATTGATTTC
HMGCS1	CATTAGACCGCTGCTATTCTGTC	TTCAGCAACATCCGAGCTAGA
HMGCR	TGATTGACCTTTCCAGAGCAAG	CTAAAATTGCCATTCCACGAGC
MVK	GGAGCAAGGTGATGTCACAAC	CGGCAGATGGACAGGTATAAGT
PMVK	CCTTTCGGAAGGACATGATCC	TCTCCGTGTGTCACTCACCA
MVD	GGACCGGATTTGGCTGAATG	CCCATCCCGTGAGTTCCTC
IDI1	TCCATTAAGCAATCCAGCCGA	CCCAGATACCATCAGACTGAGC
FDFT1	ACTTCCCAACGATCTCCCTTG	CCCATTCTCCGGCAAATGTC
SQLE	CTCCAAGTTCAGGAAAAGCCTGG	GAGAACTGGACTCGGGTTAGCT
LSS	GCACTGGACGGGTGATTATGG	TCTCTTCTCTGTATCCGGCTG
CYP51A1	ATAACCCAGCATCAGGGGAAA	CACAGTGGGAAAGTATCCATCAA
HSD17B7	TGGGATCATGCCTAATCCACA	CCAGTTCCCGAATCAGGATAAAA
DHCR7	GCAGGGGTTGTGAACAAGTAT	GAGACGGCATAGCCAAGGAT
DHCR24	GCCGCTCTCGCTTATCTTCG	GTCTTGCTACCCTGCTCCTT
SREBF2	AACGGTCATTCACCCAGGTC	GGCTGAAGAATAGGAGTTGCC
MBTPS1	CTTTCGTTCCCTCAAGTATGCT	GGGACGTGATGATTGCCACT
MBTPS2	TGGACTGTCGTCTACCTGACC	AGCAGTTTGCCATCTTATGTGG
INSIG1	CCTGGCATCATCGCCTGTT	AGAGTGACATTCCTCTGGATCTG
LDLR	GAATCTACTGGTCTGACCTGTCC	GGTCCAGTAGATGTTGCTGTGG
CDH1	GAAAGCGGCTGATACTGACC	CGTACATGTCAGCCGCTTC
CDH2	TGTTTGACTATGAAGGCAGTGG	TCAGTCATCACCTCCACCAT
SNAI1	CTTCCAGCAGCCCTACGAC	CGGTGGGGTTGAGGATCT
TWIST1	AGCTACGCCTTCTCGGTGT	CCTTCTCTGGAAACAATGACATC
TWIST2	TCCAGCAACTCCGAGAGC	GCCTCTCGAGCTCCTCCT
ZEB1	CAGTCCCACACGACCACA	GTAATGGGCCACCACCAG
ZEB2	CGAGAAAGGGCACTTGGA	CTCGTCGCCATCCTGTCT
MMP2	AGCGAGTGGATGCCGCCTTTAA	CATTCCAGGCATCTGCGATGAG
GAPDH	GTCTCCTCTGACTTCAACAGCG	ACCACCCTGTTGCTGTAGCCAA
miR-181a	AACAUUCAAC GCUGUCGGUGAGU	AACAUUCAACGCUGUCGGUGAGU
miR-103	AGCAGCAUUGUACAGGGCUAUGA	AGCAGCAUUGUACAGGGCUAUGA
3'UTR of *SREBF2*	CTAGTTGTTTAAACGAGCTCGC	ATTACACGGCGATCTTGC
pmirGLO vector	AGATCGCCGTGTAATCCACCAGGC TCAGCCCAC	CGTTTAAACAACTAGTGGGTGGTAC CAATCTGTG

Table S2. List of antibodies

Antibodies	Source	Catalog#	RRID
anti-SREBP2	abcam	ab30682	AB_779079
anti-ACAT1	Cell Signaling Technology	44276S	AB_2799262
anti-SQLE	Cell Signaling Technology	40659S	N/A
anti-HMGCR	abcam	ab242315	AB_2928124
anti-DHCR7	Novus Biologicals	NBP1-80740	N/A
anti-DHCR24	Cell Signaling Technology	2033S	AB_2091448
anti-CDH2	Thermo Fisher Scientific	TA503933	SCR_008452
anti-SNAI1	Invitrogen	14-9859-82	AB_2898546
anti-TWIST1	Cell Signaling Technology	69366S	AB_2891135
anti-MMP2	Cell Signaling Technology	40994T	AB_2799191
anti-GAPDH	Proteintech	10494-1-AP	AB_2263076
anti-GP100	abcam	ab787	AB_306146
anti-mouse IgG antibody	Santa Cruz Biotechnology	sc-516102	AB_2687626
anti-rabbit IgG antibody	Santa Cruz Biotechnology	sc-2357	AB_628497
anti-mouse- Alexa Fluor 488	Thermo Fisher Scientific	A28175	SCR_008452)
